# A systematic review of economic evaluations of cervical cancer screening methods

**DOI:** 10.1186/s13643-022-02017-z

**Published:** 2022-08-09

**Authors:** Thatohatsi Sefuthi, Lungiswa Nkonki

**Affiliations:** 1 Division of Health Systems and Public Health, Global Health Department, Faculty of Medicine and Health Sciences, Stellenbosch University, Stellenbosch, South Africa; 2 Health Economics, Division of Health Systems and Public Health, Global Health Department, Faculty of Medicine and Health Sciences, Stellenbosch University, Stellenbosch, South Africa

## Abstract

**Objective:**

The aims of this systematic review were to (1) identify primary- and model-based economic evaluations of cervical cancer screening methods and to (2) provide a contextual summary of valuation outcomes associated with three types of cervical cancer screening tests: visual inspection with acetic acid, human papillomavirus deoxyribonucleic acid, and Papanicolaou smear.

**Introduction:**

Cervical cancer screening is an important public health priority with the potential to improve the detection of precancerous lesions in high-risk females for early intervention and disease prevention. Test performance and cost-effectiveness differ based on the specific screening method used across different platforms. There is a need to appraise existing economic evaluations of cervical cancer screening methods.

**Methods:**

This review considered primary-based and model-based full economic evaluations of cervical cancer screening methods. The evaluation methods of interest included cost-effectiveness analysis, cost-utility analysis, cost-minimization analysis, cost–benefit analysis, and cost-consequence analysis. We searched Scopus, PubMed, National Health Economic Evaluation Database (NH EED), Cochrane, and the Health Economic Evaluation Database for full economic evaluations of cancer screening methods. No formal date restrictions were applied. Model-based and primary-based full economic evaluations were included. A critical appraisal of included studies was performed by the main investigator, while a second independent reviewer assessed critical appraisal findings for any inconsistencies. Data were extracted using a standardised data extraction tool for economic evaluations. The ultimate outcomes of costs, effectiveness, benefits, and utilities of cervical cancer screening modalities were extracted from included studies, analysed, and summarised.

**Results:**

From a total of 671 screened studies, 44 studies met the study inclusion criteria. Forty-three studies were cost-effectiveness analyses, one study reported both cost-utility and cost-effectiveness outcomes, and another study reported cost utilities of cervical cancer screening methods only. Human papillomavirus (HPV) DNA testing was reported as a dominant stand-alone screening test by 14 studies, while five studies reported visual inspection with acetic acid (VIA) as a dominant stand-alone screening test. Primary HPV screening strategies were dominant in 21 studies, while three studies reported cytology-based screening strategies as the dominant screening method.

**Conclusions:**

Existing evidence indicates that HPV-based and VIA testing strategies are cost-effective, but this is dependent on setting. Our review suggests the limited cost-effectiveness of cytology-based testing, which may be due in part to the need for specific infrastructures and human resources.

**Systematic review registration:**

PROSPERO CRD42020212454.

**Supplementary Information:**

The online version contains supplementary material available at 10.1186/s13643-022-02017-z.

## Background

Cervical cancer is a common malignancy and a leading cause of cancer-related mortality worldwide [1]. Cervical cancer is an essential contributor to the disease burden in sub-Saharan Africa, with an estimated 75,000 new cases documented each year and approximately 50,000 new deaths recorded annually [2]. Countries in western, middle, and southern Africa are hardest hit by cervical cancer-related deaths, with world age-standardised mortality rates of 23.0%, 21.1.%, and 20.0%, respectively [3]. The economic burden of cervical cancer is substantial. For example, a study by Wu et al. (2020) reported that, in the Henan province of China, costs associated with cervical cancer, from diagnosis to 1 year after discharge, ranged from US $8,066 to 22,888 per patient [4].

Cervical cancer is caused by infection with high-risk serotypes of the human papillomavirus (HPV) [5]. Infection with HPV can lead to the development of precancerous lesions and malignancy if left untreated [6]. Since neoplastic transformation can take years or even decades to occur, early detection and treatment of precancerous lesions provide a vital intervention opportunity [7]. The World Health Organization (WHO) has identified cervical cancer as a potentially eliminable form of cancer [7]. However, cervical cancer remains underdiagnosed in clinical settings, particularly in developing countries [8]. Evidence indicates that adequate screening reduces cervical cancer-related deaths [9]. In the global strategy for cervical cancer elimination, the WHO estimates that cervical cancer can be eliminated within this century, if, by 2030: (a) 90% of girls are fully vaccinated with the HPV vaccine by age 15, (b) 70% of women are screened with using a high-performance test at 35 years of age and 45 years of age, and (c) 90% of women with precancer are treated and 90% of women with invasive cancer managed [10]. However, an HPV vaccine global market study reported that, as of 2021, only 13% of girls are fully vaccinated and protected from cervical cancer [11]. Such data underscores the need to maintain high cervical cancer screening rates in eligible populations.

Screening for cervical cancer can be performed using unaided visual inspection with acetic acid (VIA), assisted cytological (e.g. a Papanicolaou (Pap) smear), and molecular (e.g. HPV DNA testing) methods [12, 13]. A Pap test is a liquid cytology-based test that analyses cervix cells [14]. Unaided VIA is carried out by observing cervix cell colour changes in response to acetic acid exposure [15]. These screening methods differ in their diagnostic value, accuracy, and associated costs to both the user and healthcare system [16].

Health economic evaluations [17] are comparative analyses of alternative courses of action regarding their costs and consequences [18]. They provide a framework to assist decision-makers in providing much-needed interventions based on available clinical evidence leveraged against the cost to the healthcare sector [19].

Economic evaluations from limited-resource settings like India [20] and South Africa [21] suggest that VIA is the most cost-effective primary screening test for cervical cancer. On the other hand, studies carried out in high-income countries such as Canada suggested that HPV DNA testing is the most cost-effective screening method, perhaps due in part to the ability and willingness of the country to pay for its routine adoption [22].

However, health economic evaluations focused on cervical cancer screening are limited by their use of different methodologies, and generalisation across prior studies is often not possible. The lack of consistent methods highlights the need for a methodical approach to exploring systematic differences across various economic evaluations.

We conducted an initial search of common research databases (PROSPERO, Medline, Cochrane, JBI) to identify prior studies which reviewed cervical cancer screening health economic evaluations. At least three previous systematic reviews [23–25] have provided evidence supporting the cost-effectiveness of cervical cancer screening. However, Nahvijou et al. (2014) [26] limited their systematic review to cost-effectiveness analyses of cervical cancer screening methods. In 2015, Mendes et al. [25] used mathematical models to evaluate the impact of cervical cancer screening strategies. Although critical insights were gleaned from this review, restricting the study type to mathematical modelling resulted in excluding primary-based economic evaluations. In their more recent review, Mezei et al. (2017) [24] also limited their review to cost-effectiveness analyses, focusing on lower- to middle-income countries.

Furthermore, the authors selected only model-based economic evaluations for review, thus excluding a large body of economic evaluation evidence from randomised controlled trials and primary cost-effectiveness studies. The authors did not carry out an appraisal of the methodological quality of the studies, which reduced the validity of the results. Lastly, the authors focus on the cost-effectiveness of screening methods. The present review builds on the findings reported by Nahvijou et al. (2014), Mendes et al. (2015), and Mezei et al. (2017) by evaluating all full economic evaluation methods, including cost-utility, cost–benefit, cost-minimisation, and cost-consequence analysis.

The aim of the present review was to critically appraise cervical cancer screening methods towards the improvement of precancerous lesion detection from a societal perspective, i.e. encompassing perspectives from the patient and their family members, healthcare providers, and third-party payers, and society at large.

We conducted [27] a preliminary search of PROSPERO, Medline, the Cochrane Database of Systematic Reviews, and the Joanna Briggs Institute (JBI) Database of Systematic Reviews and Implementation Reports. We found no current or underway systematic reviews on the topic. The study protocol was registered in PROSPERO under the registration number: CRD42020212454.

### Review question

From the societal perspective, what evidence does full economic evaluations provide to support the use of specific cervical cancer screening methods to improve the detection of precancerous cervical lesions in women?

### Inclusion criteria

#### Participants

The participants of interest were women eligible to be screened for cervical cancer. Eligibility criteria differed between countries.

#### Intervention(s)

We reviewed studies exploring the cost-effectiveness of three different cervical cancer screening methods, i.e. HPV testing, VIA, and cytological testing. Information on costs and outcomes was sought for the screening methods implemented as a stand-alone intervention and within the context of a broader strategy or intervention, where cervical screening was combined with HPV vaccination.

#### Comparator(s)

This review considered studies which compared the three primary methods amongst themselves and/or compared to no screening.

#### Outcomes

The review considered studies which included the following outcomes: costs, effectiveness, benefits, and utilities. These measures include uptake, coverage, incremental cost-effectiveness ratios, cost per quality-adjusted life year (QALY), and cost per disability-adjusted life year (DALY). Outcomes were extracted from the included studies.

#### Context

The review focused on full economic evaluations of cervical cancer screening methods performed without considering sociocultural, geographic, or ethnic factors.

#### Types of studies

The review considered primary- and model-based full economic evaluations of cervical cancer screening methods.

## Methods

The review was conducted using the JBI methodology for systematic reviews of economic evaluation evidence [27].

### Search strategy

The principal investigator (TS) performed a formal screening of the available academic literature from 07 September, 2020, to 18 January, 2021, across selected databases of interest (PubMed, Scopus, Cochrane, and the National Health Economic Evaluation and Health Economic Evaluation Databases). Other researchers duplicated all searches and screening of suitable studies to ensure a unanimous selection of appropriate economic evaluations for this review. The search terms used were “economic evaluation” and *cervical cancer screening* (see Additional file 1: Appendix I). All logical synonyms and iterations of these search combinations were considered depending on the database and information source. The reference lists of selected studies were also screened to identify article citations of possible interest for the present research. Inclusion criteria were as follows: (1) studies published in English and (2) studies which considered female patients screened for cervical cancer using visual (VIA), cytological (Papanicolaou smear), or molecular (HPV DNA testing) methods. Exclusion criteria were as follows: (1) studies not available in English and (2) other systematic reviews and meta-analyses. We applied no date restrictions.

All relevant citations identified using these criteria were collated and uploaded into a Microsoft Excel template, and duplicates were removed. Two independent researchers then screened titles and abstracts. Suitable studies were retrieved, and their citation details were imported into the JBI System for the Unified Management, Assessment, and Review of Information (JBI SUMARI) (JBI, Adelaide, Australia) [27]. The full-text versions of eligible studies were assessed. The reasons for the exclusion of studies were also documented and reported. The Preferred Reporting Items for Systematic Reviews and Meta-analyses (PRISMA) flow diagram was used to illustrate the flow of information through the different phases of the present review [28].

### Economic evaluation outcomes of interest

Full economic evaluation methods of interest included cost-effectiveness (CEA), cost utilities (CUA), cost–benefit (CBA), cost-minimization and cost-consequence (CC). Measures of interest included ICERS of cost/year lives saved (YLS), cost/death averted, cost/CIN2 detected, cost/QALY gained, cost/life-year (LY), marginal cost/case detected, and cost/life-year gained (LYG). Since the focus was on economic evaluations of global screening methods, no specific sociodemographic or cultural factors were considered outcomes of interest.

### Information sources

Searched databases included Scopus, HEED, NHEED, Cochrane Library, and PubMed.

### Assessment of methodological aspects of the study

The methodological quality of suitable studies was scored using the JBI standardised critical appraisal instrument [27] as well as Drummond’s checklist for assessing economic evaluations [19], which may be found in Additional file 1: Appendix III. Model-based studies were appraised using a model assessment checklist developed by Phillips et al. [29], which may be found in Additional file 1: Appendix IV.

An independent reviewer assessed critical appraisal findings for any discrepancies. We resolved disagreements were resolved through discussion. Primary-based studies were included if they scored over 5 points in the appraisal, while model-based studies were included if they scored ten and above.

### Data extraction

One reviewer extracted data from studies selected for inclusion in the review using the standardised data extraction tool from JBI SUMARI. A second independent reviewer assessed extracted data for inconsistencies and discrepancies. The JBI SUMARI tool was augmented by a data extraction tool developed by Wijnen et al. [30]. Extracted information included (1) descriptive data about cervical cancer screening studies, including study perspective, geographical setting, and study population characteristics, as well as study methods; (2) resource use results, cost and measures of cost-effectiveness, cost utility, cost–benefit, cost minimisation, and cost consequence; and (3) conclusions about factors which drive (impede) the cost-effectiveness of cervical cancer screening. Incremental cost-effectiveness ratios (ICERS) were converted to international dollars using the base year of 2020. Original costs were converted to the local currency of the study market using market exchange rate data [31]. Adjustment for inflation was carried out by multiplying ICERs by a GDP deflator obtained from the World Bank.

#### Data synthesis

Extracted data were analysed and summarised to respond to the review question using the JBI Dominance Ranking Matrix (DRM). Data analysis considered the collected data on study features, results, and authors’ conclusions about the contextual factors that drove or impeded cost-effectiveness. The DRM has three potential outcomes for the cost of intervention of interest against the health outcome(s) of interest:

- Strong dominance is characterised by decisions distinctly favouring either the intervention or comparator from a cost or clinical effectiveness standpoint.
- In weak dominance, data favours either costs or effectiveness.
- Non-dominance is characterised by a less effective or more costly intervention.

The analysis also summarised data on the characteristics, results, and authors about the circumstances in which the intervention was likely to have a higher (or less) cost–benefit, cost utility, or cost consequence.

## Results

### Study inclusion

**Fig. 1:**
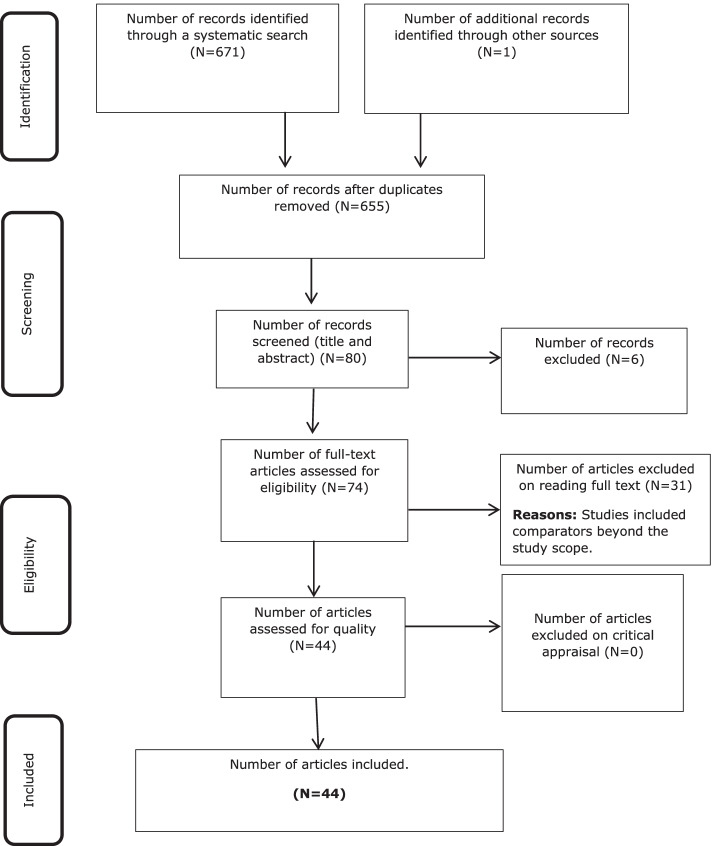
PRISMA flow diagram: search results, study selection, and inclusion process

From a total of 671 titles and citations screened following the removal of duplicates (*n* = 16), 80 abstracts were screened, and 74 studies were selected for full-text screening. Following the exclusion of ineligible studies (Fig. 1), 44 studies were included in this review.

In general, studies that were excluded during full-text selection compared health technologies beyond the scope of the research question. Additional file 1: Appendix IV documents studies ineligible following the full-text review.

### Methodological quality: primary-based studies

**Fig. 2:**
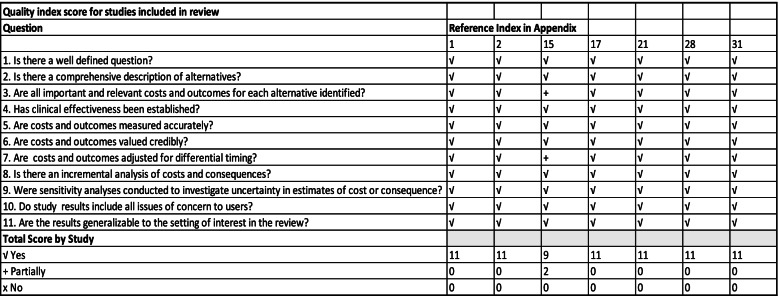
Methodological quality appraisal of primary studies

Primary-based studies were scored against eleven questions from the JBI standardised critical appraisal instrument [25] and Drummond’s checklist for assessing economic evaluations. All (*n* = 7) primary-based studies scored 11 out of 11 on the appraisal questions, except for a study by Jin et al. (2016), which had partially provided the relevant costs and outcomes for identified alternatives and had partially valued costs and consequences. Figure 2 summarises the scores of studies measured against the appraisal checklist.

### Methodological quality: model-based studies

Using a model assessment checklist developed by Phillips et al. [28], 37 studies were scored and assessed against twenty-two questions. The checklist assessed and categorised specific model elements like the present, unclear, or absent. All (*n* = 37) studies had a statement of the decision problem or objective and a statement of scope or perspective. The rationale for the model structure was provided by 97% (*n* = 36) of the studies.

Model structural assumptions were provided by 95% (*n* = 35) of the studies. All (*n* = 37) studies reported intervention strategies or comparators and the types of models they used. The model time horizon was reported by 73% (*n* = 27) of the studies, and 97% (*n* = 36) reported model disease states or pathways. Cycle length was present in 43% (*n* = 16) studies, absent in 38% (*n* = 14) studies, and unclear in 19% (*n* = 7) studies. In total, 97% (*n* = 36) studies reported both data identification and modelling elements, while 3% (*n* = 1) did not report on these elements. Baseline data was reported by 95% (*n* = 35) of the studies and was absent in 5% (*n* = 2) of the studies. Treatment effects were reported in 97% (*n* = 36) of the studies, while one treatment effects were absent in 3% (*n* = 1) of the studies. Intervention costs were reported by 97% (*n* = 36) of studies and were absent in 3% (*n* = 1) of studies. In addition, 97% (*n* = 36) of the studies reported quality-of-life weights. Data incorporation into models was reported in 97% (*n* = 36) of studies and was absent in 3% (*n* = 1) of studies.

The assessment of methodological uncertainty was reported in 78% (*n* = 29) of the studies, while 22% (*n* = 8) did not report having assessed methodological uncertainty. The studies reported structural uncertainty of models by 57% (*n* = 21), while 43% (*n* = 16) did not report structural uncertainty. Heterogeneity uncertainty was reported by 14% (*n* = 5) of studies, while 86% (*n* = 32) of the studies did not account for heterogeneity uncertainty.

The assessment of parameter uncertainty was reported in 78% (*n* = 29) of studies and was absent in 19% (*n* = 7) of the studies. It was unclear whether parameter uncertainty had been assessed in 3% (*n* = 1) of the studies. Approximately, 97% (*n* = 36) of the study models demonstrated internal consistency, while internal consistency was unclear in 3% (*n* = 1) of the studies. Models were externally consistent in 89% (*n* = 33) of the studies, while model external consistency was unclear in 11% (*n* = 4) of the studies. Figure 2 and Table 1 summarises the study scores.

**Table 1:** Model-based studies methodological quality appraisal

Quality index score for studies included in review																																					
Model Element		Reference Index in Appendix																																			
	3	4	5	6	7	8	9	10	11	12	13	14	16	18	19	20	22	23	24	25	26	27	29	30	32	33	34	35	36	37	38	39	40	41	42	43	44
1. Statement of decision problem/objective	√	√	√	√	√	√	√	√	√	√	√	√	√	√	√	√	√	√	√	√	√	√	√	√	√	√	√	√	√	√	√	√	√	√	√	√	√
2. Statement of scope/perspective	√	√	√	√	√	√	√	√	√	√	√	√	√	√	√	√	√	√	√	√	x	x	√	√	√	√	√	√	√	√	√	√	√	√	√	√	√
3. Rationale for structure	√	√	√	√	√	√	√	√	√	√	√	√	√	√	x	√	√	√	√	√	x	√	√	√	√	√	√	√	√	√	√	√	√	√	√	√	√
4. Structural assumptions	√	√	√	√	√	√	√	√	√	√	√	√	√	√	√	√	√	√	√	√	x	√	√	√	√	√	√	√	√	√	√	√	√	√	x	√	√
5. Strategies/Comparators	√	√	√	√	√	√	√	√	√	√	√	√	√	√	√	√	√	√	√	√	√	√	√	√	√	√	√	√	√	√	√	√	√	√	√	√	√
6. Model type	√	√	√	√	√	√	√	√	√	√	√	√	√	√	√	√	√	√	√	√	√	√	√	√	√	√	√	√	√	√	√	√	√	√	√	√	√
7. Time horizon	√	x	√	x	√	√	√	√	√	x	√	x	x	√	x	√	√	√	√	√	x	x	√	√	x	√	√	√	√	√	√	√	√	√	x	√	√
8. Disease states or pathways	√	√	√	√	√	√	√	√	√	√	√	√	√	√	√	√	√	√	√	√	x	√	√	√	√	√	√	√	√	√	√	√	√	√	√	√	√
9. Cycle length	+	+	√	√	+	+	√	√	+	+	x	x	x	x	x	x	√	x	x	x	x	x	x	x	+	√	√	√	√	√	√	√	√	√	x	√	√
10. Data identification	√	√	√	√	√	√	√	√	√	√	√	√	√	√	√	√	√	√	√	√	x	√	√	√	√	√	√	√	√	√	√	√	√	√	√	√	√
11. Data modelling	√	√	√	√	√	√	√	√	√	√	√	√	√	√	√	√	√	√	√	√	x	√	√	√	√	√	√	√	√	√	√	√	√	√	√	√	√
12. Baseline data	x	√	√	√	√	√	√	√	√	√	√	√	√	√	√	√	√	√	√	√	x	√	√	√	√	√	√	√	√	√	√	√	√	√	√	√	√
13. Treatment effects	√	√	√	√	√	√	√	√	√	√	√	√	√	√	√	√	√	√	√	√	x	√	√	√	√	√	√	√	√	√	√	√	√	√	√	√	√
14. Costs	√	√	√	√	√	√	√	√	√	√	√	√	√	√	√	√	√	√	√	√	x	√	√	√	√	√	√	√	√	√	√	√	√	√	√	√	√
15. Quality of life weights	√	√	√	√	√	√	√	√	√	√	√	x	√	√	√	√	√	√	√	√	√	√	√	√	√	√	√	√	√	√	√	√	√	√	√	√	√
16. Data incorporation	√	√	√	√	√	√	√	√	√	√	√	√	√	√	√	√	√	√	√	√	x	√	√	√	√	√	√	√	√	√	√	√	√	√	√	√	√
17. Assessment of methodological uncertainty	x	√	√	√	x	√	√	√	√	x	x	x	√	√	x	√	√	x	√	√	√	√	√	√	√	x	√	√	√	√	√	√	√	√	√	√	√
18. Assessment of structural uncertainty	x	√	√	√	x	x	x	x	x	x	x	x	√	x	x	√	√	x	√	√	x	x	√	√	√	x	√	√	√	√	√	√	√	√	x	√	√
19. Assessment of heterogeneity uncertainty	x	x	x	x	x	x	x	x	x	x	x	x	x	x	x	√	x	x	x	x	x	x	√	√	x	x	√	√	x	x	x	x	x	x	x	x	x
20. Assessment of parameter uncertainty	x	√	√	√	√	x	√	√	√	√	√	√	√	√	x	x	√	√	+	√	x	x	√	√	√	x	√	√	√	√	√	√	√	√	√	√	√
21. Internal consistency	√	√	√	√	√	√	√	√	√	√	√	√	√	√	√	√	√	+	√	√	+	√	√	√	√	+	√	√	√	√	√	√	√	√	√	√	√
22. External consistency	√	√	√	√	√	√	√	√	√	√	√	+	√	√	√	√	√	+	√	√	+	√	√	√	√	+	√	√	√	√	√	√	√	√	√	√	√
**Total Score by Study**																																					
√ Present	16	19	21	20	18	18	20	20	19	17	18	15	19	19	15	20	21	16	19	20	5	16	21	21	19	16	22	22	21	21	21	21	21	21	17	21	21
+ Unclear	1	2	0	1	1	1	0	0	1	2	0	2	1	0	1	0	0	2	1	0	2	0	0	0	1	2	0	0	0	0	0	0	0	0	0	0	0
x Absent	5	1	1	1	3	3	2	2	2	3	4	5	2	3	6	2	1	4	2	2	15	6	1	1	2	4	0	0	1	1	1	1	1	1	5	1	1

### Critical appraisal of results

All 44 initial studies identified were selected for inclusion in the review. Primary-based studies met the decision rules to include studies which scored above 5 using the checklist. All 37 model-based studies were included. We made an executive decision to include one study by Campos et al. (2012) [32], which had not met the decision rule since data about the model had been reported in a supplementary file.

### Characteristics of included studies

Studies were available in English and published between 2004 and 2021 (Additional file 1: Appendix V). Thirty-eight studies (88%) were model based and thus focused on hypothetical female cohorts as eligible participants. Studies were conducted across different locations, including South Africa, India, Greece, Lebanon, and Nicaragua. Although studies assumed various names to characterise perspectives, perspectives can be broadly categorised into three modalities, i.e. payer, patient, and societal perspectives. A total of 14 (33%) studies assumed a societal approach, while 18 (42%) studies used a payer perspective. The main characteristics of the studies included in the review are reported in Additional file 1: Appendix IV.

### Main findings

The most common economic evaluations examined cost-effectiveness (*n* = 43; 97%), followed by cost utility (*n* = 2.5%). A total of 20 (45%) cost-effectiveness studies reported singular screening methods as dominant, while 26 cost-effectiveness studies reported screen and treatment strategies as dominant.

### Economic evaluation findings from cost-effectiveness studies

Due to significant methodological and structural heterogeneity, results were not suitable for meta-analysis, which was further impeded by varying study designs, methodology, and outcome reporting formats. For example, no model-based studies shared the same modelling assumptions. Table 2 details the dominant stand-alone screening technologies and strategies reported in cost-effectiveness analysis studies. VIA was the dominant screening method in five studies, while HPV DNA testing was reported as the dominant screening strategy in 14 studies. No study reported cytological testing as a dominant stand-alone screening methodology for cervical cancer.

**Table 2:** Dominant stand-alone screening technology

Dominant standalone screening technology			
**Study**	**VIA**	**HPV DNA testing**	**Cytology**
Legood et al. 2005 [20]	X		
Xie et al. 2017 [33]	X		
Campos et al. 2015 [34]	X		
Lince- Deroche et al. 2015 [35]	X		
Chauhan et al. 2020 [36]	X		
Shi et al. 2011 [37]		X	
Campos et al. 2015 [34]		X	
Sharma et al. 2016 [38]		X	
Kim et al. 2005 [39]		X	
Cromwell et al. 2021 [22]		X	
Termrungruanglert et al. 2017 [40]		X	
Zhao et al. 2019 [41]		X	
Gamboa et al. 2018 [42]		X	
Jansen et al. 2020 [43]		X	
Ma et al. 2019 [44]		X	
Sroczynski et al. 2020 [45]		X	
Goldie et al. 2005 [8]		X	
Campos et al. 2018 [21]		X	
Campos et al. 2012 [46]		X	

Table 3 outlines the screening strategies which were reported as dominant. Twenty-one studies reported HPV DNA-based screening strategies as dominant, and three studies reported cytology-based screening strategies as dominant. Within the context of screening strategies, no studies reported VIA-based screening strategies as dominant.

**Table 3:** Dominant screening strategy

Dominant screening strategy				
**Study**	**HPV based**	**Cytology based**	**VIA based**	**Other**
De Kok et al. 2012 [47]	X			
Campos et al. 2014 [34]	X			
Pista et al. 2019 [48]	X			
Skroumpelos et al. 2019 [49]				X
Termrungruanglert et al. 2019 [50]	X			
Vassilakos et al. 2019 [51]	X			
Campos et al. 2018 [21]	X			
Mezei et al. 2018 [52]	X			
Lew et al. 2018 [53]	X			
Barre et al. 2017 [54]	X			
Campos et al. 2017 [55]	X			
Jin et al. 2016 [56]	X			
Burger et al. 2012 [57]		X		
Flores et al. 2010 [58]		X		
Sroczynski et al. 2011 [45]	X			
Kim et al. 2005 [39]	X			
Sherlaw-Johnson et al. 2004 [59]	X			
Chow et al. 2010 [60]	X			
Campos et al. 2012 [46]	X			
Beal et al. 2014 [61]	X			
Tantinamit et al. 2019 [62]				X
Vale et al. 2021 [63]	X			
Berkhof et al. 2010 [64]	X			
Vanni et al. 2011 [65]	X			
Lew et al. 2016 [37]	X			
Felix et al. 2016 [66]		X		

Table 4 outlines outcome measures associated with dominant screening methods and strategies. Estimated outcomes used in the cost-effectiveness analyses were as follows: ICERS of cost/year lives saved (YLS), cost/death averted, cost/CIN2 detected, cost/life year (LY), marginal cost/case detected, and cost/life-year gained (LYG). Studies which analysed both cost-effectiveness and cost utility included cost/QALY gained as an outcome measure. Costs were reported in international dollars, using the base year of 2020.

**Table 4:** Cost-effectiveness analyses results

**Study**	**Dominant screening technology/method**	**Outcome measure**	**I$ (2020)**
Legood et al. 2005 [20]	VIA	Cost/positive case detected	482.84
Xie et al. 2017 [33]	VIA	Cost/positive case detected	1,448.04
Campos et al. 2015 [34]	VIA at LTFU 60%	Cost/YLS	311.94
	VIA at LTFU 40%	Cost/YLS	181.96
Deroche et al. 2015 [35]	VIA	Cost/positive case detected	13.67
Chauhan et al. 2020 [36]	VIA	Cost/QALY gained	772.86
Shi et al. 2011 [37]	Clinician provided careHPV @ 0.5 pg/ml	Cost/YLS	2,879.31
Campos et al. 2015 [67]	HPV DNA testing at LTFU 10%	Cost/YLS	233.95
Sharma et al. 2016 [38]	HPV DNA testing every 5 years	Cost/YLS	1,355,400.48
Kim et al. 2005 [39]	HPV triage (in the Netherlands)	Cost/YLS	4,596.13
	HPV triage (in France)	Cost/YLS	3,414.27
	HPV triage (Italy)	Cost/YLS	1,969.77
Cromwell et al. 2021 [22]	HPV DNA testing every 4 years	Cost/CIN2 detected	
Campos et al. 2015 [34]	CareHPV (cervical sampling) (in India)	Cost/YLS	138.76
	CareHPV (cervical sampling) (in Nicaragua)	Cost/YLS	3,744.45
	CareHPV (cervical sampling) (in Uganda)	Cost/YLS	8,930.80
Termrungruanglert et al. 2017 [50]	hrHPV testing every 5 years	Cost/positive case detected	1,410.04
Zhao et al. 2019 [41]	CareHPV DNA testing every 3 or 5 years	Cost/positive case detected	3,038.76
Gamboa et al. 2018 [68]	HPV DNA testing every 5 years	Cost/YLS	3,119.19
Jansen et al. 2020 [69]	hrHPV testing	Cost/YLG	13,578.30
	hrHPV testing	Cost/QALY gained	15,242.86
Ma et al. 2019 [44]	HPV DNA testing every 5 years	Cost/YLS	7,690.48
	HPV DNA testing every 3 years	Cost/YLS	10,122.28
Sroczynski et al. 2010 [70]	HPV DNA testing every 2 years	Cost/YLG	138,829.99
Goldie et al. 2005 [8]	HPV DNA testing (in Kenya)	Cost/YLS	56,318.49
	HPV DNA testing (in India)	Cost/YLS	283.16
	HPV DNA testing (in Peru)	Cost/YLS	644.44
	HPV DNA testing (in South Africa)	Cost/YLS	744.64
	HPV DNA testing (in Thailand)	Cost/YLS	602.77
Campos et al. 2018 [71]	HPV DNA testing every 2 years	Cost/YLS	2,848.58
**Study**	**Dominant screening strategy**	**Outcome measure**	**I$ (2019)**
de Kok et al. 2012 [47]	Primary HPV screening	Not reported	
Campos et al. 2014 [46]	HPV-DNA screening every 5 years followed by cryotherapy (screen and treat)	Cost/YLS	21,511.43
Pista et al. 2019 [48]	HPV testing with HPV 16/18 genotyping and cytology triage	Cost/CIN2 detected	17,403.27
Skroumpelos et al. 2019 [49]	Primary HPV16/18 genotyping every 3 years	Cost/death averted	1,637,776.08
Termrungruanglert et al. 2019 [50]	HPV primary screening triage with p16/Ki-67	Cost/detected case	1,660.20
Vassilakos et al. 2019 [51]	Self-HPV testing followed by Pap testing	Cost/QALY gained	12,678.37
Campos et al. 2018 [21]	HPV testing followed by cryotherapy	Cost/YLS	13,924.77
Mezei et al. 2018 [52]	Community based self-collected HPV DNA testing followed by VIA triage	Cost/YLS	10,673.49
Lew et al. 2018 [53]	HPV testing and HPV 16/18 genotyping every 5 years	Not reported	Not reported
Barre et al. 2017 [54]	Primary HPV testing and HPV 16/18 genotyping every 5 years	Coslt/LY	2,674.12
Campos et al. 2017 [67]	HPV DNA testing followed by cryotherapy	Cost/YLS	27,288.02
Jin et al. 2016 [56]	Primary HPV DNA testing followed by followed by cytology for HPV-positive women. Testing every 5 years	Marginal cost/case detected	170,305.76
Burger et al. 2012 [57]	Unvaccinated women: cytology followed by switching to HPV testing at 34 every 4 years	Cost/YLS	23,743.81
	Vaccinated women: cytology followed by switching to HPV testing at 31, every 6 years	Cost/YLS	65,500.18
Flores et al. 2010 [58]	Pap and clinician-HPV test (30–80 years)	Not reported	
Sroczynski et al. 2011 [45]	HPV triage,1 year, age: 30 years; prior Pap, 1 year	Cost/LYG	222,752.67
Kim et al. 2005 [39]	UK: combination testing, 5 years	Cost/YLS	
Sherlaw-Johnson et al. 2004 [59]	HPV triage with LBC, 5 years	Cost/YLS	6,324.82
	Primary HPV with LBC, 5 years	Cost/YLS	7,671.45
	Combined cytology and HPV with LBC, 5 years	Cost/YLS	46,663.86
	Combined cytology with LBC, 3 years	Cost/YLS	780,481.31
Chow et al. 2010 [60]	HPV testing followed by Pap smear triage every 5 years	Cost/QALY gained	2,940.98
Campos et al. 2012 [46]	Primary HPV-based testing strategies	Cost/YLS	584.88
Beal et al. 2014 [61]	hrHPV with molecular triage	Cost/prevented missed case	580.76
Tantitamit et al. 2019 [62]	HPV genotyping with reflex dual stain cytology	Cost/QALY gained	837.20
Vale et al. 2021 [63]	hrHPV testing with LBC triage	Cost/detection of CIN2/3	36.26
Berkhof et al. 2010 [64]	HPV DNA testing every 5 years with cytology triage	Cost/QALY gained	25,783.00
Vanni et al. 2011 [65]	HPV DNA testing followed by cytology triage every year	Cost/YLS	770.83
Lew et al. 2016 [53]	5-yearly HPV screening with partial genotyping for HPV16/18 and referral to colposcopy and cytological triage of other oncogenic types	Not reported	X
Felix et al. 2016 [66]	Co-testing using LBC and HPV 16 18/45 genotyping	Cost/QALY gained	2,550.16

### Economic evaluation findings from cost-utility studies

Guerrero et al. [72] compared VIA to Pap smear screening implemented alone or with HPV vaccination at different coverages. Outcome measures were ICERS in the form of cost/QALY gained and reduction in cervical cancer. VIA was associated with the highest dominance and cost-saving in various coverage scenario analyses, with ICERS ranging from dominant to 1443 USD. VIA augmented by HPV vaccination of pre-adolescent girls was reported to be dominant at a coverage of 80%, with an ICER of US $783. Zhao et al. (2019) performed a cost-effectiveness analysis of cervical cancer screening methods, augmented by a utility analysis. The authors found that careHPV testing every 5 years had the highest cost-utility ratio (1,783.8 Yuan/year) [41].

## Discussion

We critically appraised economic evaluation studies of cervical cancer screening methods (*n* = 44). In total, 44 studies (100%) supported the cost-effectiveness of cervical cancer screening. Our results suggested that primary HPV DNA testing strategies are cost-effective in several settings. VIA may be cost-effective in some environments, including rural areas, but not in others. Similarly, cost-utility findings comparing cytology and VIA often describe that VIA has higher utility. These findings are echoed by Mezei et al. (2017). After performing a systematic review of the cost-effectiveness of cervical cancer screening methods in LMICs, they concluded that HPV testing and VIA were the most cost-effective screening methods [24]. Pap testing is frequently dominated by HPV testing and VIA but is cost-effective in co-testing and triaging. Our results also suggest that cervical cancer screening modalities are most effective when applied within a broader context of treatment and intervention. This would include consideration of the health economics of cervical cancer in addition to evidence for the effectiveness of different established modalities. Our review further suggests that sample collection, screening sequence and algorithms, and coverage are essential.

One factor that influences the cost-effectiveness of cervical cancer screening modalities is sample collection. Mezei et al. [52] compared self-collection followed by clinic-based VIA triage to clinic-based collection and triage in HPV-positive females in Uganda. The reduction in cervical cancer incidence and ICERs (USD/YLS) was used as cost-effectiveness measures. The use of Monte Carlo modelling allowed the authors to show that self-collection was more cost-effective than clinic-based VIA triage-based ICER outcomes. Using cytology-based screening as a comparator, Vassilakos et al. [51] also reported that offering HPV self-testing is more cost-effective compared to cytology and associated with a reduction in cervical cancer cases and cancer-related mortality. Both authors correlate a critical gain to HPV self-testing is increased population coverage.

The method sequence could also affect cervical cancer screening cost-effectiveness. Jin et al. [56] compared the three screening methods for cervical cancer of interest in this review and found significant differences in their diagnostic accuracy. Co-testing was identified as more accurate but also less cost-effective. These findings echo those reported by Campos et al. [55], who compared different methods and interventions in their lifetime risk reduction and ICERS (USD/YLS). These measures found HPV testing with intervention to be more cost-effective compared to cytology-based strategies. Using the Nicaraguan cost-effectiveness threshold (GDP per capita of US $2090), HPV cryotherapy remained comparatively cost-effective, with an ICER of US $320/YLS [55].

Several studies included in this review underscored the importance of screening coverage. In Lebanon, results from a model-based cost-effectiveness analysis indicated that using cytology as a screening modality with a shift from the current 20% coverage to at least 50% would reduce cervical cancer incidence considerably [38]. More gains would be achieved if HPV testing was used as a screening modality, at 50% coverage, resulting in a 23.4% reduction in the incidence of cervical cancer [38]. Modulating coverage for different strategies (50–80%) tend to favour the cost-effectiveness of HPV-based screening strategies [38].

Several study limitations should be noted. None of the included studies which used models and simulations accounted for uncertainty associated with heterogeneity, and few accounted for model structural uncertainty. Consequently, internal or external model consistency could not be guaranteed. Several model-based studies used the same model Campos et al. [71 46 34]. Consequently, study findings are not disparate. Lastly, critical appraisal and data extraction were performed by one reviewer. However, this limitation was offset by critical appraisal and extracted data being assessed for inconsistencies by another independent reviewer.

## Conclusions

In conclusion, our review supports the general cost-effectiveness of HPV testing and VIA as screening strategies for cervical cancer. Compared to HPV testing and VIA, cytology testing is the least cost-effective. Future studies would do well to examine the health economics of cervical cancer screening, with emphasis on the test performance of different screening modalities. Furthermore, parameters such as the order of screening methods, and its relationship to the screening intervention, screening coverage, screening modality, and the number of screening visits, could have important implications for care. The ultimate success of cervical cancer screening and treatment could depend on a broader perspective in deciding which strategy is most appropriate for the individual patient and context.

### Study implications for practice, policymakers, and future researchers

This review sought to synthesise available evidence on cervical cancer screening methods and strategies to achieve optimal precancerous lesion detection and thus avert cervical cancer. Given the significant heterogeneity of studies included in our review, study results could not be pooled and were not suitable for meta-analyses, a limitation common to economic evaluation systematic reviews. This limitation underscores the need to develop and further standardise economic reporting. An interim measure which researchers can apply is sub-set group analysis, i.e. aim to pool and compare studies similar in setting, participants, and outcomes. Ultimately, researchers should keep in mind that health economic reviews are not intended to provide conclusive recommendations for routine practice but rather to guide policymakers in developing optimised strategies for testing and intervention [27].

Review findings have demonstrated the multi-faceted nature required to achieve optimal screening strategies. An extension of existing research might show the need for clinicians to offer due consideration to the individual and public health costs of cervical cancer screening. HPV and VIA screening might be more appropriate screening options for clinicians. A combined approach might also prove feasible, and clinicians might need to consider the order in which screening is performed in order to maximise cost-effectiveness. Furthermore, a large body of models and simulations targeted towards cervical cancer screening evaluation exist. Countries intending to introduce more relevant and improved cancer strategies can leverage the existing body of knowledge by learning from documented best practices.

#### Recommendations for research

Few studies have discussed how HPV vaccination could inform decisions on screening reduction, which is vital as several countries seek to roll out HPV vaccination. It will be essential to know what bearing this will have on cervical cancer screening programmes to minimise inefficiencies. Further research would do well to determine what treatment options are associated with ideal clinical and economic value.

## Supplementary Information

**Additional file 1: Appendix I**: Search strategy. **Appendix II**: Data extraction instrument. **Appendix III**: JBI standardised tool and Drummond's Checklist . **Appendix IV**: Phillip et al Checklist for Model-Based Studies. **Appendix V**: Studies excluded on full text. **Appendix VI**: Characteristics of Included Studies. Table: Characteristics of Included Studies - Economic Evaluation Form. **Appendix VII**: Abstract Checklist.

## Abbreviations

VIA: Visual inspection with acetic acid
HPV DNA: Human papillomavirus deoxyribonucleic acid
Pap smear: Papanicolaou smear
WHO: World Health Organization
HIV: Human immunodeficiency virus
HPV: Human papillomavirus
HERC: Health Economics Resource Centre
BIA: Budget impact analysis
LEEP: Loop electrosurgical excision procedure
ICER: Incremental cost-effectiveness ratio
CIN2 +: Cervical intraepithelial neoplasia
PEPFAR: President’s Emergency Plan for AIDS Relief
NH EED: National Health Economic Evaluation Database
HEED: Health Economic Evaluation Database
JBI DRM: Joanna Briggs Institute Dominance Ranking Matrix
ASMR: Age-standardised mortality rates
JBI: Joanna Briggs Institute
GDP: Gross domestic product
