# Supplementary material for: A systematic review of economic evaluations of cervical cancer screening methods
Source: Syst Rev. 2022 Aug 9;11:162. doi: 10.1186/s13643-022-02017-z (PMC9361672; doi:10.1186/s13643-022-02017-z)
Supplement: Supplementary file 1 — Additional file 1: Appendix I: Search strategy. Appendix II: Data extraction instrument. Appendix III: JBI standardised tool and Drummond's Checklist . Appendix IV: Phillip et al Checklist for Model-Based Studies. Appendix V: Studies excluded on full text. Appendix VI: Characteristics of Included Studies. Table: Characteristics of Included Studies - Economic Evaluation Form. Appendix VII: Abstract Checklist. [file 13643_2022_2017_MOESM1_ESM.pdf]

Appendices

Appendix I: Search strategy

Pubmed Search

Search conducted on the 7th September 2021

Search String

(((uterine cervical neoplasm [MeSH Terms]) OR (cervical cancer [Title/Abstract] OR uterus cancer [Title/Abstract] OR cervix cancer [Title/Abstract] OR uterine cancer [Title/Abstract] OR cervical carcinoma [Title/Abstract] OR cervical neoplasm [Title/Abstract]))

AND

((early detection of cancer [MeSH Terms]) OR (screening [Title/Abstract] OR cervical screening [Title/Abstract] OR medical examination [Title/Abstract] OR mass screening [Title/Abstract] OR early detection of cancer [Title/Abstract] OR diagnosis [Title/Abstract] OR diagnostic screening programs [Title/Abstract])))

AND

((Cost-Benefit Analysis [MeSH Terms]) OR (economic evaluation [Title/Abstract] OR economic appraisal [Title/Abstract] OR economic valuation [Title/Abstract] OR economic assessment [Title/Abstract] OR cost-effectiveness analysis [Title/Abstract] OR cost benefit analysis [Title/Abstract]))

Cochrane Search

Search Name:

Date Run: 06/09/2021 22:14:16

Comment:

ID Search Hits

#1 MeSH descriptor: [Uterine Cervical Neoplasms] explode all trees (2183)

#2 (cervical cancer OR uterus cancer OR cervix cancer OR uterine cancer OR cervical carcinoma OR cervical neoplasm):ti,ab,kw (7426)

#3 MeSH descriptor: [Early Detection of Cancer] explode all trees (1309)

#4 (early detection of cancer OR screening OR cervical screening OR medical examination OR mass screening OR early detection of cancer OR diagnosis OR diagnostic screening programs):ti,ab,kw (208527)

#5 MeSH descriptor: [Cost-Benefit Analysis] explode all trees (7324)

#6 (Cost-Benefit Analysis OR economic evaluation OR economic appraisal OR economic valuation OR economic assessment OR cost-effectiveness analysis OR cost benefit analysis):ti,ab,kw (27762)

#7 #1 OR #2 (7792)

#8 #3 OR #4 (208527)

#9 #5 OR #6 (27762)

#10 #7 AND #8 AND #9 (128)

SCOPUS Search

Search conducted on the 7 September 2021.

(TITLE-ABS-KEY ("cervical cancer" OR " cancer of the cervix" OR "uterine cervix" OR "uterine cancer" OR "cervical carcinoma" OR "uterine cervical neoplasms") AND TITLE-ABS-KEY ("screening" OR "cervical screening" OR "medical examination" OR "mass screening" OR "early detection of cancer" OR "diagnosis" OR "diagnostic screening programs") AND TITLE-ABS-KEY ("economic evaluation" OR "economic appraisal" OR "economic valuation" OR "economic assessment" OR "cost-effectiveness analysis" OR "cost benefit analysis")) AND (LIMIT-TO (OA , "publisherfullgold"))

NHSHEED Search

Search conducted on the 12th of November 2020.

(("Uterine Cervical Neoplasms"[Mesh]) AND "Early Detection of Cancer"[Mesh]) AND "Cost-Benefit Analysis"[Mesh]

NHEED Search

Search conducted on the 12th of November 2020.

(("Uterine Cervical Neoplasms"[Mesh]) AND "Early Detection of Cancer"[Mesh]) AND "Cost-Benefit Analysis"[Mesh]

Database Screenshots

Pubmed

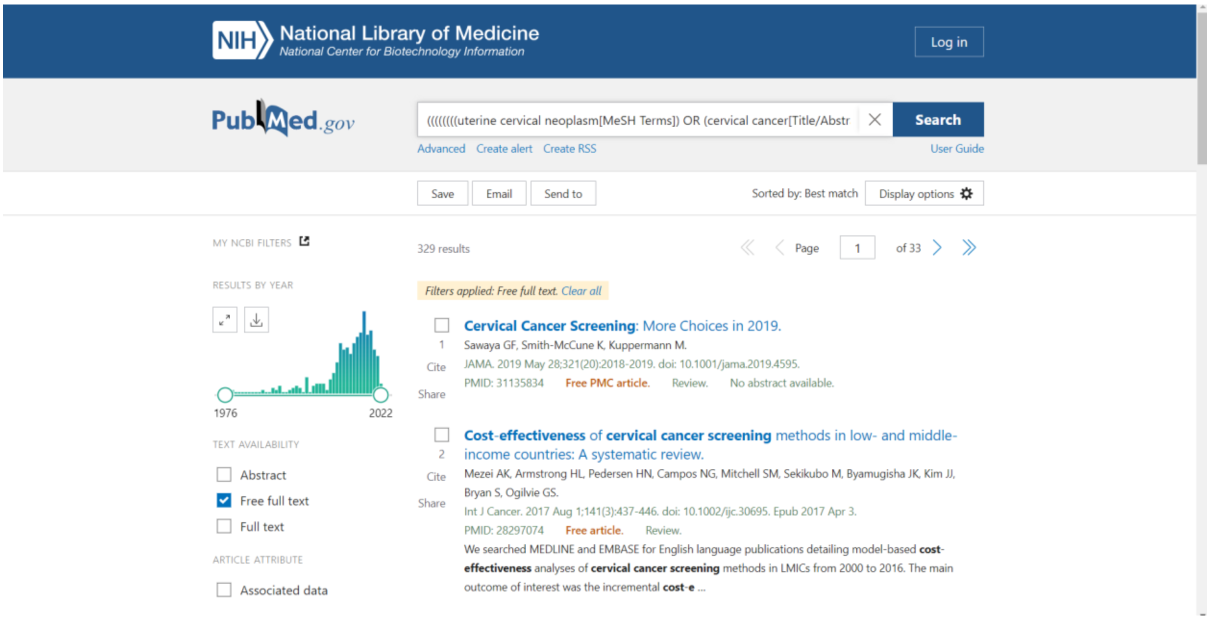

Cochrane Library

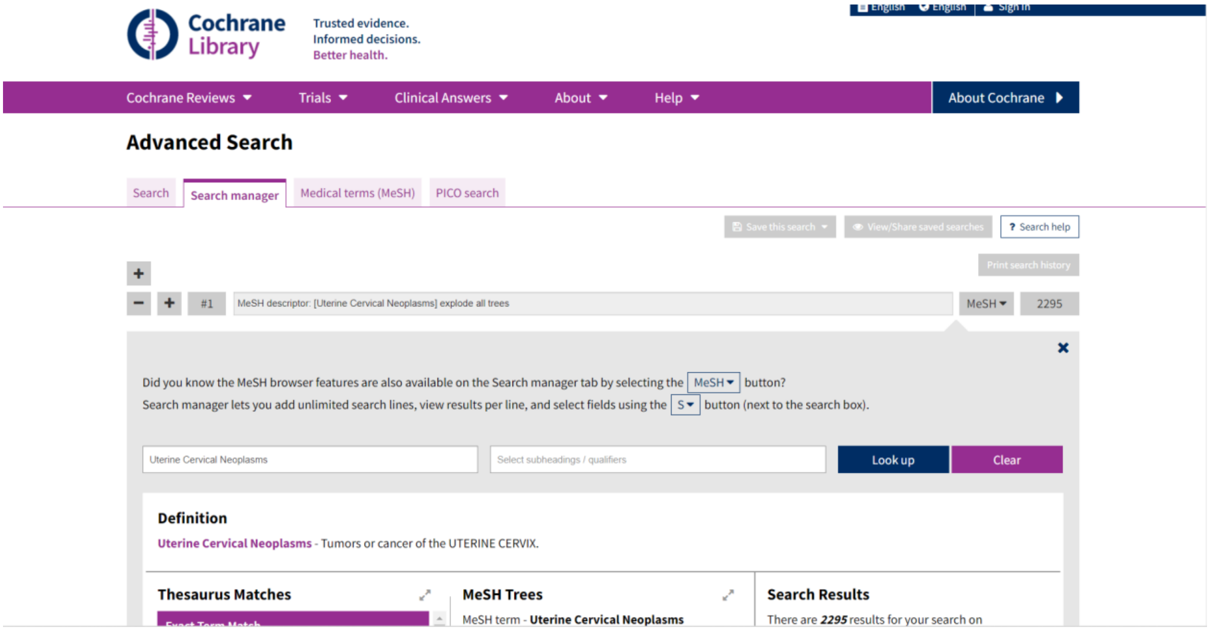

Scopus

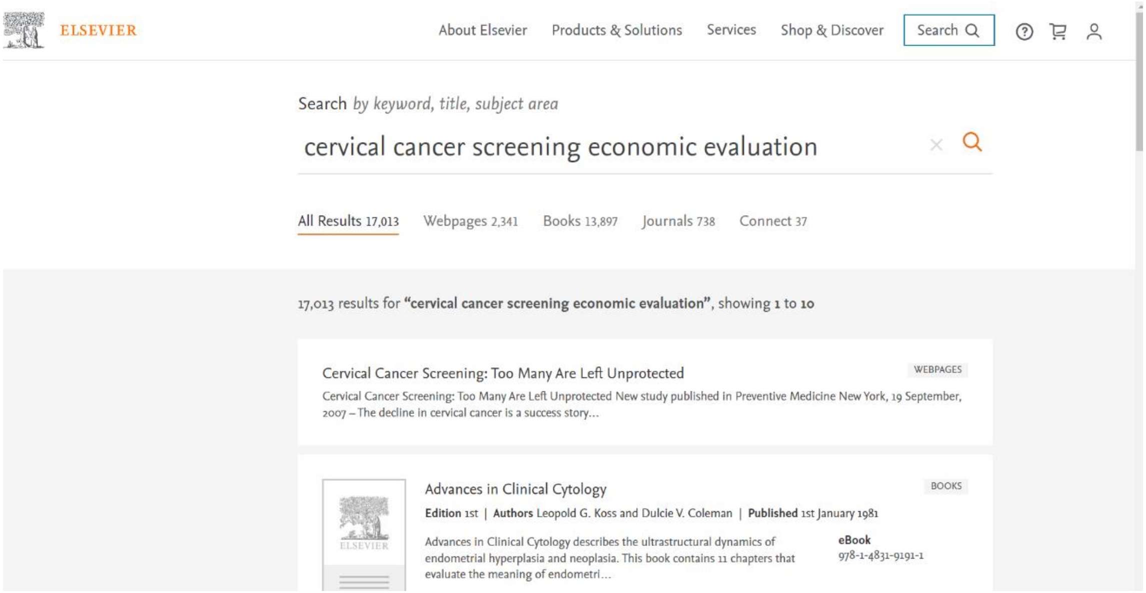

NHSHEED

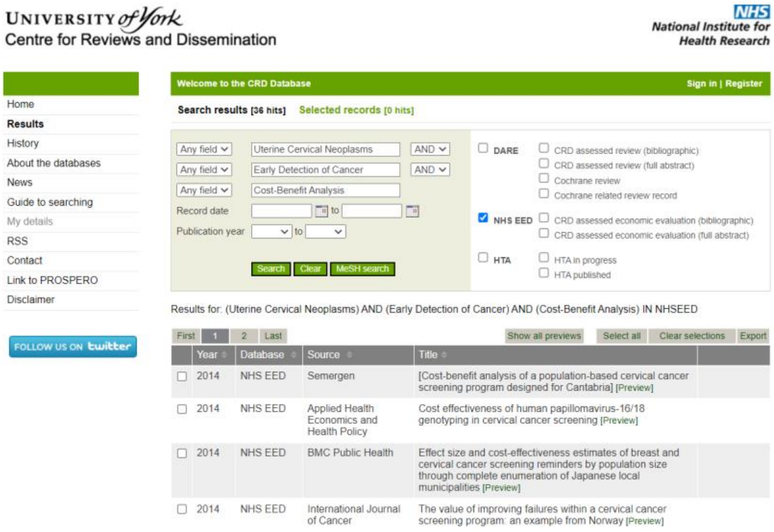

NHEED

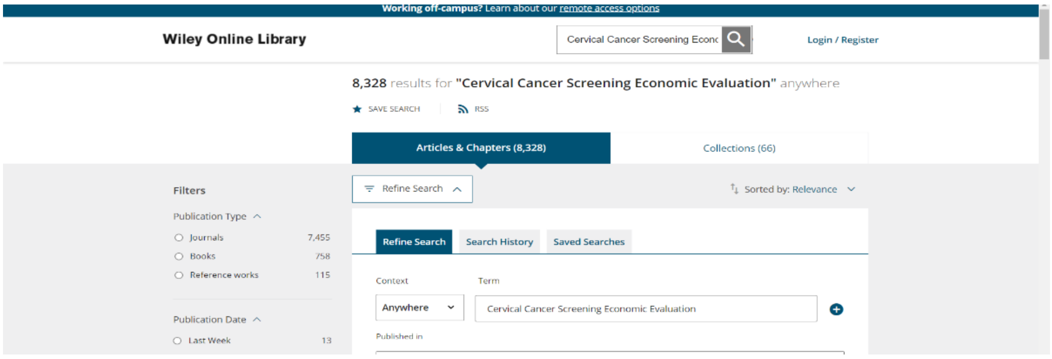

Appendix II: Data extraction instrument

**Review title: A Systematic Review of Economic Evaluations of Cervical Cancer Screening Methods**	
**Review Variables**	
1. Reviewer	2.
3. Date	4.
5. Year	6.
7. Author	8.
9. Record Number	10.
11. Sources of funding	12.
13. Competing interests	14.
15. Publication Type	16.
17. Method of Evaluation	18.
19. Study Perspective	20.
21. Intervention	22.
23. Comparator	24.
25. Setting	26.
27. Geography	28.
29. Participants	30.
31. Source of effectiveness data	32.
33. Authors conclusions	34.
35. Reviewers comments	36.
**Clinical Effectiveness Results**	
37. Study design	38.
39. Year range of primary studies	40.
41. Analysis used	42.
43. Data source of effects	44.
45. Method of measurements of effects	46.
47. Method of valuation of effects	48.
49. Clinical outcome results	50.
**Economic Effectiveness Results**	
51. Dates of economic data	52.
53. Modelling used	54.
55. Measure of benefits used in economic evaluation	56.
57. Direct costs	58.
59. Indirect costs	60.
61. Currency	62.
63. Time frame of analysis	64.
65. Discount rate	66.
67. Discount rate for costs	68.
69. Discount rate for effects	70.
71. Inflation rate	72.
73. Reference year	74.
75. Methods for identifying resource use	76.
77. Assumptions for the measurement of resources	78.
79. Methods used to calculate unit costs	80.

81. Statistical Analyses	82.
83. Estimated benefits uses in EE	84.
85. Cost results	86.
87. Synthesis of costs and results	88.
89. Analysis of uncertainty outcomes of analysis of sensitivity analysis	90.
91. Outcomes of analysis of sensitivity analysis	92.

Appendix III: JBI standardised tool and Drummond's Checklist

	Questions
1	Is there a well-defined question?
2	Is there comprehensive description of alternatives?
3	Are all important and relevant costs and outcomes for each alternative identified?
4	Has clinical effectiveness been established?
5	Are costs and outcomes measured accurately?
6	Are costs and outcomes valued credibly?
7	Are costs and outcomes adjusted for differential timing?
8	Is there an incremental analysis of costs and consequences?
9	Were sensitivity analyses conducted to investigate uncertainty in estimates of cost or consequence?
10	Do study results include all issues of concern to users?
11	Are the results generalizable to the setting of interest in the review?

Appendix IV: Phillip et al Checklist for Model-Based Studies

Model Element	Present	Absent	Unclear
Statement of decision problem/objective			
Statement of scope/perspective			
Rational for structure			
Structural assumptions			
Strategies/Comparators			
Model type			
Time horizon			
Disease states or pathways			
Cycle length			
Data identification			
Data modelling			
Baseline data			
Treatment effects			
Costs			
Quality of life weights			
Data incorporation			
Assessment of uncertainty			
Methodological uncertainty			
Structural uncertainty			
Heterogeneity uncertainty			
Parameter uncertainty			
Internal consistency			
External consistency			

Appendix V: Studies excluded on full text

1. Khushalani JS, Trogdon JG, Ekwueme DU, Yabroff KR. Economics of public health programs for underserved populations: a review of economic analysis of the National Breast and Cervical Cancer Early Detection Program.

Reason for exclusion: Article does not compare cervical screening methods. Article describes Economic analyses and types of analyses.

2. Messoudi W, Elmahi T, Nejjari C, Tachfouti N, Zidouh A, Saadani G, Moriña D,Diaz M. Cervical cancer prevention in Morocco: a model-based cost-effectiveness analysis.

Reason for exclusion: Compares VIA to no screening at all or to HPV vaccination.

3. de Kok IMCM, Korfage IJ, van den Hout WB, Helmerhorst TJM, Habbema JDF, Essink-Bot ML, van Ballegooijen M. Quality of life assumptions determine which cervical cancer screening strategies are cost-effective.

Reason for exclusion: Evaluates quality of life assumptions that that are used to calculate cost-effectiveness in screening.

4. Campos NG, Tsu V, Jeronimo J, Mvundura M, Kim JJ. Estimating the value of point-of-care HPV testing in three low- and middle-income countries: a modelling study.

Reason for exclusion: Measures cost-effectiveness of HPV (only) screening at various coverages

5. Li Y, Carlson E, Villarreal R, Meraz L, Pagán JA. Cost-effectiveness of a patient navigation program to improve cervical cancer screening.

Reason for exclusion: Evaluates cost-effectiveness of patient navigation programs, not screening methods.

6. Mezei AK, Armstrong HL, Pedersen HN, Campos NG, Mitchell SM, Sekikubo M, Byamugisha JK, Kim JJ, Bryan S, Ogilvie GS. Cost-effectiveness of cervical cancer screening methods in low- and middle-income countries: A systematic review.

Reason for exclusion: Study is a systematic review.

7. Vodicka EL, Babigumira JB, Mann MR, Kosgei RJ, Lee F, Mugo NR, Okech TC, Sakr SR, Garrison LP Jr, Chung MH. Costs of integrating cervical cancer screening at an HIV clinic in Kenya.

Reason for exclusion: No: Costing study and is therefore a partial economic evaluation, not full.

8. Pedersen K, Sørbye SW, Burger EA, Lönnberg S, Kristiansen IS. Using Decision-Analytic Modelling to Isolate Interventions That Are Feasible, Efficient and Optimal: An Application from the Norwegian Cervical Cancer Screening Program.

Reason for exclusion: Compares cytology to colposcopy.

9. Huh WK, Williams E, Huang J, Bramley T, Poulios N. Cost-effectiveness of human papillomavirus-16/18 genotyping in cervical cancer screening.

Reason for exclusion: Intervention being evaluated is HPV-genotyping.

10. Nahvijou A, Hadji M, Marnani AB, Tourang F, Bayat N, Weiderpass E, DaroudiR, Sari AA, Zendehdel K. A systematic review of economic aspects of cervical cancer screening strategies worldwide: discrepancy between economic analysis and policymaking.

Reason for exclusion: Systematic Review

11. Berkhof J, Bogaards JA, Demirel E, Diaz M, Sharma M, Kim JJ. Cost-effectiveness of cervical cancer prevention in Central and Eastern Europe and Central Asia.

Reason for exclusion: Evaluates the cost-effectiveness of adding HPV vaccination and does not compare screening methods.

12. Kim JJ, Campos NG, O'Shea M, Diaz M, Mutyaba I. Model-based impact and cost-effectiveness of cervical cancer prevention in sub-Saharan Africa.

Reason for exclusion: Examines cost-effectiveness of HPV vaccination.

13. Esselen KM, Feldman S. Cost-effectiveness of cervical cancer prevention.

Reason for exclusion: Methods paper describing Cost-effectiveness analysis.

14. Della Palma P, Moresco L, Giorgi Rossi P. Health technology assessment report: Computer-assisted Pap test for cervical cancer screening.

Reason for exclusion: No English version found.

15. de Bekker-Grob EW, de Kok IM, Bulten J, van Rosmalen J, Vedder JE, Arbyn M,Klinkhamer PJ, SiebersAG, van Ballegooijen M.Liquid-based cervical cytology using Thin Prep technology: weighing the pros and cons in a cost-effectiveness analysis.

Reason for exclusion: No alternative screening comparators.

16. Vokó Z, Nagyjánosi L, Margitai B, Kövi R, Tóth Z, László D, Kaló Z.Modeling cost-effectiveness of cervical cancer screening in Hungary.

Reason for exclusion: Singular screening method (Pap smear testing) with or without colposcopy.

17. Obradovic M, Mrhar A, Kos M. Cost-effectiveness analysis of HPV vaccination alongside cervical cancer screening programme in Slovenia.

Reason for exclusion: Compares the CEA of adding vaccination to one screening modality (Pap smear testing)

18. Dee A, Howell F.A cost-utility analysis of adding a bivalent or quadrivalent HPV vaccine to the Irish cervical screening programme.

Reason for exclusion: Compares the cost-effectiveness of adding vaccination to screening.

19. Chuck A. Cost-effectiveness of 21 alternative cervical cancer screening strategies.

Reason for exclusion: Comparators beyond scope

20. Ratushnyak, S., Hoogendoorn, M., van Baal, P.H.M.Cost-Effectiveness of Cancer Screening: Health and Costs in Life Years Gained.

Reason for exclusion: Does not compare cervical cancer screening strategies.

21. Sander, B., Wong, W.W.L., Yeung, M.W., Ormanidhi, O., Atkin, K., Murphy, J., Krahn, M., Deeks, S.L.The cost-utility of integrated cervical cancer prevention strategies in the Ontario setting - Can we do better?

Reason for exclusion: Provides utility and cost-effectiveness for combining screening with vaccination.

22. Vokó, Z., Nagyjánosi, L., Margitai, B., Kövi, R., Tóth, Z., László, D., Kaló, Z.Modeling cost-effectiveness of cervical cancer screening in Hungary.

Reason for exclusion: Uses irrelevant comparators- Compares cytology with colposcopy to cytology with no colposcopy.

23. De Koning, H. The cost-effectiveness of cancer screening.

Reason for exclusion: Full text not available

24. Han, L; Chang, X; Song, P; Gao, L; Zhang, Y; An, L; Shen, J. An on-going study of three different cervical cancer screening strategies based on primary healthcare facilities in Beijing China.

Reason for exclusion: Does not report any ICERS or relevant economic evaluation outcomes.

25. Kitchener, HC; Blanks, R; Cubie, H; Desai, M; Dunn, G; Legood, R; Gray, A; Sadique, Z; Moss, S. MAVARIC - a comparison of automation-assisted and manual cervical screening: a randomised controlled trial.

Reason for exclusion: Health technology intervention and comparator beyond scope of study.

26. Sankaranarayanan, R; Budukh, AM; Rajkumar, R. Effective screening programmes for cervical cancer in low- and middle-income developing countries.

Reason for exclusion: Does not provide an economic evaluation/analyses.

27. Bidus M A, Maxwell G L, Kulasingam S, Rose G S, Elkas J C, Chernofsky M, Myers E R. Cost effectiveness analysis of liquid-based cytology and human papillomavirus testing in cervical cancer screening.

Reason for exclusion: Compares the same strategy, only different sensitivities.

28. Taylor L A, Sorensen S V, Ray N F, Halpern M T, Harper D M. Cost-effectiveness of the conventional Papanicolaou test with a new adjunct to cytological screening for squamous cell carcinoma of the uterine cervix and its precursors.

Reason for exclusion: Compares Pap to Pap and Speculoscopy

29. Legood R, Gray A, Wolstenholme J, Moss S. Lifetime effects, costs, and cost effectiveness of testing for human papillomavirus to manage low grade cytological abnormalities: results of the NHS pilot studies.

Reason for exclusion: Focuses on triaging women who already have some level of CIN.

30. Diaz M, Kim JJ, Albero G, de Sanjose S, Clifford G, Bosch FX, Goldie SJ. Health and economic impact of HPV 16 and 18 vaccination and cervical cancer screening in India.

Reason for exclusion: Compares vaccination versus unvaccinated populations.

31. Ginsberg GM, Lauer JA, Zelle S, Baeten S, Baltussen R. Cost effectiveness of strategies to combat breast, cervical, and colorectal cancer in sub-Saharan Africa and South East Asia: mathematical modelling study.

Reason for exclusion: Does not specify comparators and outcomes.

Appendix VI: Characteristics of Included Studies

Table: Characteristics of Included Studies - Economic Evaluation Form

ID #	Study	Economic Evaluation Type	Study Perspective	Study Design	Participant characteristics	Study Setting/Geography	Funding Source
1	Rosa Legood et al.2005	Cost-effectiveness analysis	Health service perspective	Primary	131, 178 women aged 30-59	Nargis Dutt Memorial Cancer Hospital, India	Not declared
2	Xie et al.2017	Cost effectiveness Cost-benefit, Cost-Utility	User perspective	Primary	3086 women aged 36-65 years	Rural region in China	Institute of Cancer Prevention and Control (Wuhan, China)
3	Campos et al. 2015	Cost-effectiveness analysis	Not reported	Model-based	Hypothetical cohort of Ugandan women	Health centres and Homes, Uganda	US National Cancer Institute
4	Campos et al. 2014	Cost-effectiveness analysis	Societal perspective	Model-based	Hypothetical population of women aged 30-49 years	El Salvador	Basic Health International, Einhorn Family Charitable Trust
5	Skroumpelos et al. 2019	Cost-effectiveness analysis	Social Health Insurance perspective	Model-based	Hypothetical cohort of Greece women aged 25-65	Greece Public Health System, Greece	No funding received
6	Pista et al. 2019	Cost-effectiveness analysis	Public Healthcare Payer	Model-based	Hypothetical cohort of 2,078,039 Portugue	Portugal	Roche SistemasDiagnosticos LDA

			Perspective		se women		
7	Termrungruanglert et al. 2019	Cost-effectiveness analysis	Healthcare payer perspective	Model-based	Hypothetical cohort of 67,959 360 Thai women aged 30-65	Thailand	Roche Diagnostics
8	Vassilakos et al. 2019	Cost-effectiveness evaluation	Healthcare payers perspective	Model-based	Unscreened women aged 25 years and living in Switzerland without cervical cancer	Switzerland	Not reported
9	Campos et al.2018	Cost-effectiveness application	Societal Perspective	Model-based	Hypothetical cohort of women aged 30-65	El Salvador	Einhorn Family Charitable Trust
10	Mezei et al. 2018	Cost-effectiveness analysis	Societal Perspective	Model-based	Hypothetical cohort of Ugandan women	Rural Uganda	Canadian Institute for Health Research
11	Lew et al. 2018	Cost-effectiveness Analysis	Health services perspective	Model-based	Hypothetical cohort of Australian women aged 10-84	Australia	Department of Health, Australia
12	Barre et al. 2017	Cost effectiveness analysis	All payers perspective	Model-based	A hypothetical closed cohort of100,000 French women	France	National cancer institute

					aged 25-65		
13	Campos et al. 2017	Cost-effectiveness analysis	Societal Perspective	Model-based	Hypothetical cohort of Nicaraguan women aged 30-59 years	Nicaragua's public sector health facilities	Bill and Melinda Gates Foundation
14	Sharma et al. 2016	Cost-effectiveness analysis	Societal Perspective	Model-based	Hypothetical cohort of Lebanese women	Lebanon	Health Decision Science, Harvard T Chan School of Public Health
15	Jin et al. 2016	Cost-effectiveness analysis	Payer perspective	Primary Study Design	Retrospective cohort of 99,549 women tested from 2004-2010	Cleveland Clinic, United States of America	Cleveland Clinic Research Program Committee
16	Felix et al. 2016	Cost-effectiveness analysis	Healthcare payer’s perspective	Model-based	Hypothetical cohort of 1 million USA women aged 30-70	United States of America	Not reported
17	Lince-Deroche et al.2015	Cost-effectiveness analysis	Provider perspective	Primary-based	1,202 HIV Positive South African women aged 18-65	South Africa	USAID
18	Guerrero et al. 2015	Cost-utility analysis	Health systems perspective	Model-based	Filipino women aged 30-45, pre-adolescent Filipino	Philippines	Not reported

					girls aged 11		
19	Burger et al.2012	Cost-effectiveness analysis	Societal	Model based	Hypothetical cohort of Norwegian women	Norway	University of Oslo, The Norwegian Cancer Society, US National Cancer Institute, Bill and Melinda Gates Foundation
20	Shi et al.2011	Cost-effectiveness analysis	Societal	Model-based	Hypothetical cohort of Chinese women	Rural China	Chinese Ministry of Science and Technology
21	Flores et al. 2010	Cost-effectiveness analysis	Perspective of Mexican Institute of Social Security (IMSS) in Morelos, Mexico	Retrospective cohort	Mexican women aged 20-80	Mexico	
22	Sroczynski et al. 2011	Cost-effectiveness analysis	German Healthcare System	Model-based	Hypothetical cohort of German women aged 15 and older	Germany	German Agency for HTA
23	Kim et al. 2005	Cost-effectiveness analysis	Societal	Model-based	Hypothetical cohort of women	United Kingdom, Netherlands, France, Italy	Not reported
24	Sherlaw-Johnson. 2004	Cost-effectiveness analysis	Healthcare provider perspective	Model-based	Hypothetical cohort of women in the UK	United Kingdom	Not reported

					aged 21-64		
25	Chow et al.2010	Cost-effectiveness analysis	Department of Health perspective	Model-based	Hypothetical cohort of Taiwanese women aged 30 and above	Taiwan	"Bureau of Health Promotion, Department of Health of the Republic of China (Taiwan)
26	Campos et al. 2013	Cost-effectiveness analysis	Not reported	Model-based	Hypothetical cohorts of women eligible for screening in respective countries	Kenya, Mozambique, Tanzania, Uganda, and Zimbabwe	1. Bill and Melinda Gates Foundation 2. National Cancer Institute.
27	de Kok et al. 2012	Cost-effectiveness analysis	Not reported	Model-based	Unvaccinated eight million European women born from 1939 to 1992.	European countries	European Union, Dutch National Institute for Public Health and the Environment
28	Cromwell et al 2021	Cost-effectiveness analysis	Not reported	Primary study	19009 women aged 25-65	Canada	Canadian Institute for Health Research
29	Campos et al 2015	Cost-effectiveness analysis	Societal perspective	Model-based	Reported elsewhere	Nicaragua, Uganda, India	Bill and Melinda Gates Foundation
30	Termrungruanglert et al 2017	Cost-effectiveness analysis	Healthcare provider perspective	Model-based	Hypothetical cohort of women	Thailand	None

31	Beal et al 2014	Cost-effectiveness analysis	Not reported	Primary study	Mexican women	Mexico	Not reported
32	Tantitamit et al 2019	Cost-effectiveness analysis	Provider perspective	Model-based	hypothetical cohort of women	Thailand	Roche Diagnostics
33	Zhao et al 2019	Cost-effectiveness and Cost-utility analysis	Societal perspective	Model-based	3000 women aged 35-64	China	National Health Commission of China
34	Gamboa et al 2018	Cost-effectiveness analysis	Payer perspective	Model-based	Hypothetical cohort	Columbia	Not reported
35	Vale et al 2021	Cost-effectiveness analysis	Payer perspective	Model-based	Hypothetical cohort of women	Brazil	1.UNICAMP 2. Roche diagnostics
36	Chauhan et al 2020	Cost-effectiveness analysis	Societal Perspective	Model-based	Hypothetical cohort of women aged 30–65	India	Department of Health Research, India
37	Jansen et al 2020	Cost-effectiveness analysis	Societal perspective	Model-based	Hypothetical cohort of women	Netherlands	EU Framework Program
38	Ma et al 2019	Cost-effectiveness analysis	Societal	Model-based	Hypothetical cohort of women	China	National Health Commission of China
39	Sroczynski et al 2010	Cost-effectiveness analysis	Payer perspective	Model-based	Hypothetical cohort of women	Germany	Not reported
40	Berkhof et al 2010	Cost-effective	Payer perspective	Model-based	Hypothetical cohort of 4000	Netherlands	The Netherlands Organisation

		ness analysis			000 women		for Health Research and Development
41	Vanni et al 2011	Cost-effectiveness analysis	Health systems perspective	Model-based	Hypothetical cohort of women	Brazil	Evando Chagras Clinical Research Institute
42	Goldie et al 2005	Cost-effectiveness analysis	Not reported	Model-based	Hypothetical cohort of women	India, Kenya, Peru, South Africa, Thailand	Not reported
43	Lew et al 2016	Cost-effectiveness analysis	Health services perspective	Model-based	Hypothetical cohort of women	New Zealand	Ministry of Health, New Zealand
44	Campos et al 2018	Cost-effectiveness analysis	Societal Perspective	Model-based	Hypothetical cohort of women	South Africa	Bill and Melinda Gates Foundation

Appendix VII: Abstract Checklist

Section and Topic	Item #	Checklist item	Reported (Yes/No)
**TITLE**			
Title	1	Identify the report as a systematic review.	Yes
**BACKGROUND**			
Objectives	2	Provide an explicit statement of the main objective(s) or question(s) the review addresses.	Yes
**METHODS**			
Eligibility criteria	3	Specify the inclusion and exclusion criteria for the review.	Yes
Information sources	4	Specify the information sources (e.g. databases, registers) used to identify studies and the date when each was last searched.	Yes
Risk of bias	5	Specify the methods used to assess risk of bias in the included studies.	Yes
Synthesis of results	6	Specify the methods used to present and synthesise results.	Yes
**RESULTS**			
Included studies	7	Give the total number of included studies and participants and summarise relevant characteristics of studies.	Yes
Synthesis of results	8	Present results for main outcomes, preferably indicating the number of included studies and participants for each. If meta-analysis was done, report the summary estimate and confidence/credible interval. If comparing groups, indicate the direction of the effect (i.e. which group is favoured).	Yes
**DISCUSSION**			
Limitations of evidence	9	Provide a brief summary of the limitations of the evidence included in the review (e.g. study risk of bias, inconsistency and imprecision).	Yes
Interpretation	10	Provide a general interpretation of the results and important implications.	Yes
**OTHER**			
Funding	11	Specify the primary source of funding for the review.	YES

Section and Topic	Item #	Checklist item	Reported (Yes/No)
Registration	12	Provide the register name and registration number.	Yes

From: Page MJ, McKenzie JE, Bossuyt PM, Boutron I, Hoffmann TC, Mulrow CD, et al. The PRISMA 2020 statement: an updated guideline for reporting systematic reviews. BMJ 2021;372:n71. doi: 10.1136/bmj.n71
